# Artificial intelligence education for radiographers, an evaluation of a UK postgraduate educational intervention using participatory action research: a pilot study

**DOI:** 10.1186/s13244-023-01372-2

**Published:** 2023-02-03

**Authors:** Riaan van de Venter, Emily Skelton, Jacqueline Matthew, Nick Woznitza, Giacomo Tarroni, Shashivadan P. Hirani, Amrita Kumar, Rizwan Malik, Christina Malamateniou

**Affiliations:** 1 Department of Radiography, Faculty of Health Sciences, School of Clinical Care Sciences, Nelson Mandela University, Port Elizabeth, South Africa; 2 Division of Midwifery and Radiography, School of Health and Psychological Sciences, City, University of London, London, UK; 3 Department of Perinatal Imaging and Health, King’s College London, London, UK; 4 Guy’s and St Thomas’ NHS Foundation Trust, London, UK; 5 Radiology Department, University College London Hospitals, London, UK; 6 School of Allied and Public Health Professionals, Canterbury Christ Church University, Canterbury, UK; 7 Cit-AI, Department of Computer Science, City, University of London, London, UK; 8 BioMedIA, Department of Computing, Imperial College London, London, UK; 9 Centre for Healthcare Innovation Research, City, University of London, London, UK; 10 Frimley Health NHS Foundation Trust, London, UK; 11 Royal Bolton Hospital, Farnworth, UK; 12 Department of Radiography, HESAV University, Lausanne, Switzerland

**Keywords:** Artificial intelligence, Radiography, Education, Evaluation, Action research

## Abstract

**Background:**

Artificial intelligence (AI)-enabled applications are increasingly being used in providing healthcare services, such as medical imaging support. Sufficient and appropriate education for medical imaging professionals is required for successful AI adoption. Although, currently, there are AI training programmes for radiologists, formal AI education for radiographers is lacking. Therefore, this study aimed to evaluate and discuss a postgraduate-level module on AI developed in the UK for radiographers.

**Methodology:**

A participatory action research methodology was applied, with participants recruited from the first cohort of students enrolled in this module and faculty members. Data were collected using online, semi-structured, individual interviews and focus group discussions. Textual data were processed using data-driven thematic analysis.

**Results:**

Seven students and six faculty members participated in this evaluation. Results can be summarised in the following four themes: a. participants’ professional and educational backgrounds influenced their experiences, b. participants found the learning experience meaningful concerning module design, organisation, and pedagogical approaches, c. some module design and delivery aspects were identified as barriers to learning, and d. participants suggested how the ideal AI course could look like based on their experiences.

**Conclusions:**

The findings of our work show that an AI module can assist educators/academics in developing similar AI education provisions for radiographers and other medical imaging and radiation sciences professionals. A blended learning delivery format, combined with customisable and contextualised content, using an interprofessional faculty approach is recommended for future similar courses.

## Key points

- A novel postgraduate module on AI for radiographers was developed and evaluated.
- Blended-learning delivery, customisable and contextualised course content, and interprofessional faculty are the ways forward for an ideal AI course for radiographers.
- Future courses could use this approach to develop their own AI training.

## Background

Artificial intelligence (AI) refers to the computer systems’ ability to perform tasks ordinarily associated with human intelligence. In the medical imaging context, AI applications may apply to tasks related to visual perception, speech recognition, decision-making, and natural language processing [1]. AI is a rapidly advancing healthcare technology. It is increasingly being implemented in clinical service delivery in medical imaging and other healthcare sectors during the last decade, with an exponential increase in the last 4 years [2]. This accelerated implementation has been facilitated by, among other factors, faster processing speeds of servers, the availability of “big data” to train, test and validate AI algorithmic models and our increasing understanding of human brain learning processes, which now underpin the implementation of neural networks in deep learning applications [3, 4].

Different policy and research publications have highlighted the importance of education and upskilling of all healthcare practitioners to enable acceptance of AI and implementation into a digital future [5–11]. Radiology and radiography are amongst the most technology-enabled healthcare professions [12], which increasingly use AI not only in image interpretation and reporting but also in many operational aspects of clinical practice, such as vetting of examinations, patient positioning, image quality optimisation, image postprocessing, image reconstruction and management of workflows [1, 13–16].

Radiologists have already started to design and implement educational provisions to teach AI and to acquire the necessary knowledge and skills to efficiently and safely use AI-enabled technologies in clinical practice [17–20]. However, until very recently, the radiography profession lagged behind in formal AI educational provisions. Radiography has a tradition of adaptability to technological advancements [21, 22], harnessing the benefits and mitigating the associated risks of these technologies. Recent work has reiterated the importance of AI education, as a priority for safe and efficient clinical implementation of AI tools by radiographers, and of clinically meaningful, prospective AI research for building the evidence base [14, 23–26]. In line with previous research studies, a development and implementation of a postgraduate-level introductory module on AI for radiographers was performed [14, 23–26]. To the best of our knowledge, this is the first radiographer-specific module on AI in the Europe, Middle East and Africa (EMEA) region.

Therefore, the aim of this paper is to evaluate and discuss the first iteration of this early adopter postgraduate-level module on AI for radiographers, which has been designed and delivered in the UK and available to students in the EMEA region.

## Methods

The structure of this paper was guided by the consolidated criteria for reporting qualitative research (COREQ) [27].

### Research design

**Fig. 1:**
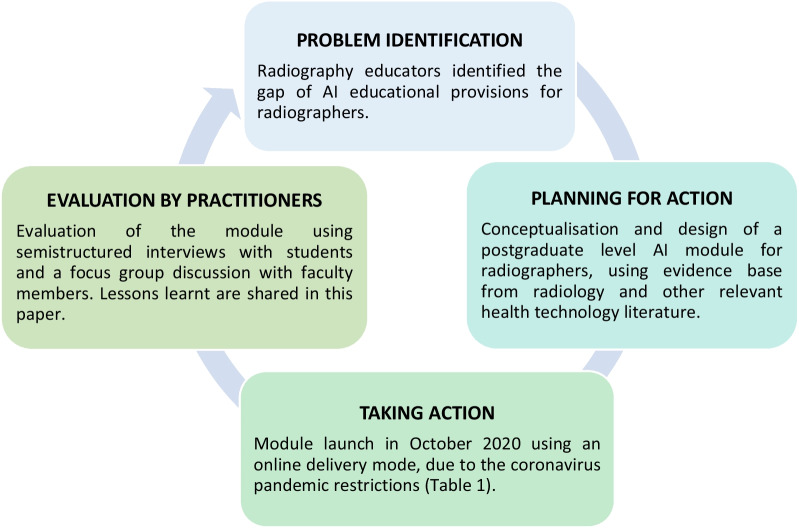
Visual summary of the participatory action research cycle, as applied in this study. This cycle can be used to continuously improve the module over time, based on student and faculty feedback

A participatory action research (PAR) approach was employed as the optimal qualitative research methodology to address a challenge, as identified by practitioners: the limited availability of AI educational provisions for radiographers [28]. The PAR cycle comprises problem identification, planning for action, taking action, evaluation and specifying learning by practitioners (clinicians or educators) [29, 30]. This design allows for an in-depth, detailed evaluation of a selected sample size before moving onto larger scale quantitative studies and enables continuous improvement of the intervention (e.g. the AI module in this case) through continuous feedback by key stakeholders (students and faculty). Figure 1 provides a visual overview of the PAR cycle and how it was applied in this study.

Radiography academics identified the limited AI education provisions for radiographers [23, 24]. To address this, they conceptualised, designed and launched a postgraduate, master’s level AI module. The module development, organisation, content and implementation were grounded on radiology and technological innovation literature [18–20, 31–34] and informed by expert perspectives gained through interdisciplinary and transdisciplinary discussions between radiographers, computer scientists, radiologists, psychologists and AI industry experts.

This postgraduate-level module was initially offered by City, University of London in October 2020 titled. Details about its structure, accreditation, aims and assessments are summarised in Table 1 and described below.

**Table 1:** Details about the AI module for radiographers

Module name	Introduction to artificial intelligence for radiographers
Level	Master’s (level 7)
Credits	30 UK credits (equivalent to 15 ECTS credits) Elective in year 2 for a 3-years part-time master’s programme in Radiography. Continuing professional development (CPD) offering was also an option, either for credits (with assessments) or non-credit bearing (without assessments)
Length	12 weeks
Mode of delivery	Online
Pedagogical approach	Flipped classroom Resources provided by thematically organising key articles, textbooks, policies, videos, podcasts and websites on the learning management system Synchronous, weekly, 2-h long live tutorials/discussions on MS Teams Asynchronous, self-directed study of articles and pre-recorded lectures and discussion board activities on the learning management system Asynchronous Online forum for student support Formative feedback for assignments to support learning Adjustments for neurodivergent students (including subtitles in videos, where feasible)
Content themes (informed by evidence [18–20, 31–34])	Basic AI concepts and terminologies Clinical applications of AI in projectional and cross-sectional imaging, reporting, ultrasound, mammography, and interventional radiology Basic computer science fundamentals underpinning algorithms and associated workshop for hands-on work Impact of AI on workflow in medical imaging Ethical considerations associated with AI Patient and healthcare acceptability of AI Industry-led workshops to introduce state-of-the-art AI applications and foster networking
Assessment strategy	Short report and presentation on two different AI-enabled tools in medical imaging

The module is aimed at recent graduates, clinical practitioners, managers, researchers and educators in radiography who wish to enhance their knowledge of the basic principles and clinical applications of AI in medical imaging. Students enrolled in this module are introduced to:

- Key concepts of AI and computer science,
- Examples of AI applications in different imaging modalities of radiography,
- Ethical considerations pertaining to responsible, transparent, equitable AI implementation and use of AI tools in medical imaging,
- AI governance and regulation,
- Acceptability and adoption of new technology by clinical practitioners,
- Impact of AI on radiography workflows,
- Evaluation and validation frameworks for AI.

A combination of didactic lectures, industry-led interactive symposia, hands-on workshops and tutorials are used to offer support with assignments and to facilitate assimilation of new concepts. Learning was assessed by one written essay and one oral presentation about the application and evaluation of different AI algorithms in a clinical context and within the students’ clinical practice.

A minimum aggregate mark of 50% is required to pass the module. All students get a certificate of completion at the end of the module. The module, worth 30 UK academic credits at postgraduate level, was costed at 1500 GBP during the first iteration and is subject to annual adjustment. Original planning was for an on-campus, in-person delivery, and over a 30-hour-long compact teaching week; however, due to the COVID-19 pandemic restrictions, the delivery of this module was adjusted to be fully online and spread in 2.5 h sessions over 12 weeks.

A formal evaluation is key to inform future implementations of any action research-based intervention and to bring about culture change in practice [35]. Input from students and faculty was sought, as vital to provide recommendations for refinement and to inform future offerings of this educational intervention [36–41].

### Ethics approval

Ethics approval for this study was granted by the City, University of London School of Health Sciences Research Ethics Committee (REC Ref: ETH2021–0948).

### Target population, sampling strategy and participant recruitment

A purposive sampling strategy was employed to recruit participants and to gain data-rich contextualised perspectives [41]. The seventeen enrolled students (*n* = 17) and eight faculty members (*n* = 8) that taught in this first running of module were invited to participate via email by the assigned research assistant (first author). During consenting, their right to decline the invitation or withdraw from the study at any point was underscored. To safeguard impartial and objective evaluation, the programme director of this postgraduate module was not involved in either participant recruitment or data collection for the study.

### Data collection

The MS Teams platform (Microsoft, United States) was used for data gathering [42]. Data from student participants were gathered using online synchronous, one-on-one, semi-structured interviews, and data from faculty members were gathered using an online, synchronous focus group discussion (FGD) [42]. Individual interviews were selected for the students to enable them to comfortably share their experiences with the interviewer, gain in-depth information and to maintain confidentiality and privacy [43]. A FGD was deemed appropriate for the faculty members, to capture their collective perspectives and offer a more holistic understanding of their experiences, which would not have been possible through individual interviewing [44].

The interviews and FGD were audio-recorded to allow for generation of a verbatim transcript for data analysis purposes. The initial interview question used for both student and faculty participants was as follows: *Tell me how you experienced the artificial intelligence module.* Individualised probing questions were then asked, based on participants’ responses to gain a deeper understanding of their narratives. The details of these questions are in Table 2. The interviews’ length ranged between 19 min and 11 s to 53 min and 2 s. The FGD lasted for 1 h and 19 min. Data collection was terminated once data saturation was achieved, i.e., no new information emerged.

**Table 2:** Examples of probing questions

*Probing questions for student participants*	
Which were the positive aspects of the module for you? Please explain any reasons	
Did you find what you learned useful in your daily clinical practice or research?	
Did you find the taught and online material sufficient?	
Did you find the taught material to be at appropriate level for your learning?	
Which were the negative aspects of the module for you? Please provide any reasons	
Did it meet your expectations?	
How did you experience the online delivery of the module?	
How can the module be improved in the future?	
Did you do the assignments? Were the assignments a useful learning experience?	
Would you prefer in-person classes, if permissible?	
What else would they like to see?	
*Probing questions for faculty participants*	
Which were the positive aspects of this module for you?	
What potential challenges have you encountered or foresee for this module?	
How the module may be further improved, in your opinion?	

### Data analysis

The auto-generated transcripts, audio-recording of the interviews and FGD were downloaded. Transcripts were compared with the audio-recordings, and any mistakes were corrected to be a true reflection of the participants’ narratives. The prepared transcript was sent separately to each participant for member-checking and approval before data analysis.

Approved transcripts were coded using an inductive, data-driven, open and descriptive coding approach. Tesch’s eight step approach to coding was applied [45, 46]. Codes attached to segments of data were interrogated and reflected on to construct themes that provide a cohesive, descriptive account of the participants’ collective experiences. Codes from both students and faculty were integrated to provide a collective narrative of all participants’ experiences. A social constructivist epistemology and relativist ontology underpinned the data analysis process. That means the researchers viewed knowledge as socially constructed from multiple, subjective realities that are products of human action and interaction within a particular context [47].

### Trustworthiness and credibility

Strategies to ensure rigour and integrity of this study were guided by the trustworthiness model of Guba and Lincoln [48]. Credibility was achieved through member-checking, where participants had to review the transcripts of their interview or FGD, suggest changes and approve the final version of the transcript as a true representation of their realities. Credibility was ascertained by verbatim transcription, discussion of the coding process and construction of themes by two researchers, as well as detailed documentation of all analysis choices by the first author in a reflective diary. These strategies also ensured dependability and confirmability of the study. Transferability and authenticity were enhanced through detailed, vivid descriptions of the findings, context and methodology, and the use of verbatim quotations from participants to substantiate interpretative descriptions [48]. The use of both interviews and FGD, as well as the curation of a reflective diary of the first author, completed after each data collection event, guided analysis and ensured the authenticity of the context for each data collection point. This data-source triangulation further enhanced trustworthiness.

## Results

Seven student participants (*n* = 7, 2 men and 5 women) and six faculty members (*n* = 6, 4 men and 2 women) comprised the final sample. Student participants to this study were all early to mid-career radiographers from varied areas of practice: clinicians, academics, researchers, managers and representatives of professional societies/associations from the UK, Denmark, Italy, Switzerland and United Arab Emirates. All participating faculty members were UK-based, experienced clinicians, academics and/or researchers from a multidisciplinary background: radiography, radiology, computer science, and psychology.

Four themes were constructed from the interview and FGD data to provide a descriptive narrative of the participants’ experiences of the AI module, either as students or faculty members. Integrated codes from the interviews and FGD are presented in Table 3 [49]. Verbatim quotations are labelled IP to refer to student Interview Participants or FGDP to refer to faculty Participants that were part of the FGD, with a number added at the end, to differentiate participants’ contributions from each other.

**Table 3:** Codes, code frequency and convergence of codes to construct the themes

Codes	Frequency (*n*)	Themes
*Student participants*		
Buffer of experience	4	Theme 1: Participants’ professional and educational background influenced their experiences
Met learning needs as an introduction	2	
*Faculty participants*		
Assume no knowledge to pitch content and learning activities	1	
Background influenced preparation/experience	1	
*Student participants*		
Mode of delivery	25	Theme 2: A meaningful learning experience
Views on assignments	14	
High quality content	15	
Knowledge gained	15	
Online interactions	9	
Module length	7	
Different didactic/pedagogical approaches	7	
Module organisation	11	
*Student participants*		
Views on assignments	14	Theme 3: Barriers to learning and threats to module status
Module length	7	
Timing—presentation	3	
Cost	2	
Certification issues	2	
Technology mediated learning as a barrier	1	
Preparation must be linked to lesson	1	
Socialisation missed	1	
*Student participants*		
Focus on how AI works	20	Theme 4: The ideal introductory AI module
Balance between cohort, intention and content is important—iterative process	3	
Selection of learning experiences need to be purposeful and not repetitive	4	
Access to materials after the module	2	
Important to include in curricula	3	
*Faculty participants*		
Focus on how AI works	18	
Balance between cohort, intention and content is important—iterative process	3	
Important to include in curricula	2	
Selection of learning experiences need to be purposeful and not repetitive	3	
Consider IPE approach for imaging professionals	5	

### Theme 1: Participants’ professional and educational background influenced their experience

Faculty participants approached their preparations for the lectures assuming students had no foundational knowledge of AI to provide more general insights about the fundamentals of AI and its clinical applications.

“…assume no knowledge and you’d have to start building up for them” – FGDP5

“…kept things quite general where we could raise those discussions…that were transferable between different technologies…modalities” – FGDP4

Considering this approach adopted by the faculty members, the student participants indicated that overall, the module met their expectations of an introductory module.

“…personally, [it] was an introduction. I knew very little…it met perfectly to the needs of what I needed” – IP2

“…it’s an introduction so maybe we should not know everything” – IP1

Student participants explained that their different needs could be ascribed to the different roles that they fulfil and their interests, that motivated their enrolment to this module.

“…wanted a bit more about certain modalities as the focus was more of ultrasound, MRI than it was in plain DR and CT examinations in my opinion, but I think…depending on which research you are leading and…role you are having” [sic.] – IP4

The above provided significant context to understand the variations of participants’ appraisals of the AI module and recommendations for future offerings.

### Theme 2: A meaningful learning experience

Participants considered their participation in the module as a meaningful learning experience as they reflected on the module delivery, organisation, content and pedagogical approaches used to mediate teaching and learning.

“…I found it…really helped me to develop my own learning” – IP2

The module delivery was regarded an enabler of learning due to flexibility, which allowed digital, synchronous and asynchronous engagement during and outside of working hours. Participants felt that this fitted well with their working schedules, since it allowed students to catch up asynchronously if they missed sessions. Participants also regarded the online delivery more cost-effective in terms of traveling and accommodation associated with campus-based delivery.

“I like to receive the lessons, the lectures and to hear it before the session, it was very nice” – IP1

“…online is great for me, there’s no additional cost to go with it…” – IP2

“…the lessons were very well scheduled, well it was at night…so most people could attend” – IP3

“…I think it was really great that you were actually able to take it in the evenings…and your busy schedule on a daily space is not blocking the opportunity to participate [sic.] – IP4

Participants felt the module was organised well in a logical and user-friendly manner on the learning management system; this and the use of a scaffolded approach in the module, from fundamentals to more advanced clinical applications, facilitated their learning. They felt there was broad coverage of the field of AI at an introductory level. The use of different experts in the related fields associated with AI in the context of radiography and different instructional methods enhanced the learning experience further. Clarification of expectations at the start of the module further contributed to a meaningful learning experience as it set the scene for the way forward for participants, who knew what to expect. This was complemented with adequate, appropriate and timely guidance from the module lead.

“…it gave us a good insight of AI from every perspective…so it was very broad [the module lead] was very clear about everything that [they] were going to do and the way things were delivered…” – IP3

“…it was very well organised. Given that it was for the first time…it was split into a few parts and the splitting was also very good [the module lead] guide me the right way [sic.]” – IP5

“…every session…or every pedagogical approach had its own…positive impact in terms of having the different information learned in a different way…” – IP7

Participants reported that the resources that were provided and the approaches used to deliver the content (discussion boards and flipped classroom, encouraging active student engagement) contributed to a positive learning experience. The assignments were perceived as extensions of student participants’ learning and gave them an opportunity for self-assessment of their comprehension of AI relative to medical imaging practice.

“…you could do pre-reading and have your questions formulated in your mind before you went to the section…interaction with the rest of the group and also the presenters at that time…live…the chat box” – IP2

“…the way in which it was assessed, the presentation and the essay it really makes you think deeply…it really makes you dig deep into that matter [chosen topic], so it was really good” – IP3

“…the level of information was really high. It gave me new insight …about how AI is used at the moment” – IP4

“…I will think is just about right, the materials they’ve put in” [sic.] – IP6

Given the positive experiences that student participants acknowledged, they strongly agreed that they would recommend the module to colleagues. They felt it empowered one with knowledge to comprehend how AI technologies operate and it also informed respective research endeavours.

“…it’s opened a lot of research in this field also” – IP1

“…this course, if anything, you know what, if you asked me things about artificial intelligence now, I do have a good base…” – IP7

### Theme 3: Barriers to learning and threats to module status

Student participants indicated that some aspects related to the module design and pedagogy, and marketing were considered to be barriers to learning and a potential threat to the module’s status. The module was offered during a time of the year that some participants felt was rather busy, although they were mindful that this may be perceived differently depending on the different roles of those enrolled. This made it difficult for some of them to fully immerse themselves with the module requirements to get the most out of it.

“…we have a lot of things…at the end of the year you know…it’s crazy” – IP1

Participants indicated a need for greater alignment in scheduling between preparations for lessons, the content of the lesson and its ultimate congruence with the expectations of the end-of-module assignments. Module length was also perceived as an exacerbating factor as some participants felt that the duration of the module, given the richness of the content, could be extended to allow them time to be prepared for the assignments, especially because this was a novel area for the participants.

“…given the nature of the information, I mean the novelty…it’s a total new concept…I needed more time” – IP7

Participants felt the digital learning and teaching space itself was a barrier, since physical contact and networking are preferred and a necessary part of their development. However, they did acknowledge this was due to the required coronavirus pandemic related restrictions at the time of delivery and that opportunities to enhance engagement among students and between students and faculty members were offered, but the physical contact would allow for more immediate interactions compared to the digital space.

“…could have been nice to meet up physically to get more network in the group…it just giving a different atmosphere when you’re looking people in the eyes” – IP4

“…you may not be able to understand because of technology…when some lecturers are delivering it, it is difficult even if you don’t understand something…in the classroom, you can easily call back, but…not possible during online” – IP6

Participants highlighted the cost of the module may be unaffordable by potential students. One student felt that better advertising of the full curriculum in detail would be an advantage for future student recruitment, as it would justify the value for money and the uniqueness of this course.

“The only issues for us is that it’s kind of pricey” – IP4

“…this certificate fails to describe the real depth of the course…people are not ready to pay this much money for an introduction…” – IP7

### Theme 4: The ideal introductory AI module

Participants reflected on characteristics of an ideal introductory AI module and made recommendations for future occurrences. The first recommendation was that the module should be flexible for a varied audience, whilst being cognisant of the context in which the module is being delivered.

“…always those contextual factors that might influence what we really want…so that you can tailor it to that target audience” – IP5

Participants also suggested that an introductory module in AI should largely focus on the fundamentals of AI to explain how it works, the concept of explainability, and examples of clinical applications. Participants felt that knowledge of AI fundamentals and of some key clinical applications would enable them to more confidently use AI in clinical practice. It was also suggested by the participants that teaching students how to engage and appraise AI literature is critical to foster their understanding of the literature and assist them to critique AI applications in practice or during procurement thereof.

“…make sure that people are knowledgeable and know how AI works” – IP2

“…something about an intro to the algorithms…a session dedicated to explainability and uncertainty” – FGDP3

“…the critical appraisal of the AI literature, because this is fast moving, it’s clickbait headlines…so I think that element could be brought in a little bit because radiographers, although may not be the core decision makers about purchasing, they will be using it and they will need to be able to critique industry proposals…” – FGDP2

Participants also felt having compulsory and elective sessions incorporated in an introductory module would be beneficial for them, so that they can customise their learning in line with their needs and preferences based on their knowledge gaps and areas of interest. They recommended the materials should be available for some time after the completion of the module, so that they can be accessible when needed.

“…it’s too short to take everything, to look at everything so if we can have a longer access, it can be nice yeah” [sic.] – IP1

“…maybe for some weeks, we’ll be doing holistically the AI application of general radiology. And tailor some lectures as it relates to the individual areas depending on your modality…” – IP6

Participants suggested that learning activities and content must be purposefully selected so as to eliminate unnecessary repetition while balancing reinforcement of learning.

“Probably one thing that I didn’t enjoy much, I think one of the speciality…was a bit repetitive…” – IP3

“…make sure that there was enough overlap to reinforce learning but not…duplication” – FGDP2

The introduction of AI in undergraduate medical radiation sciences curricula was also highlighted, so that students are being prepared from an earlier stage for their future career. While acknowledging the interprofessional faculty, participants agreed that the ideal course should have a strong interprofessional education (IPE) approach, since AI occurs within an ecosystem with other healthcare professionals.

“AI is progressing … and my students need to be informed…looking into adding in our curriculum components that touch base on artificial intelligence” – FGDP7

“…we’re making a demarcation between radiology and radiographers, other aspects of imaging, whereas actually the truth is, very little about any of these tools is specific to any of our roles” – FGDP1

Participants indicated that a student-led, synchronous discussion forum using videoconferencing could extend their learning through peer-to-peer informal teaching to foster a sense of community and allow for further networking, beyond the one already established within the course.

“…let’s say, right, once every week…if anybody wants to drop in and have a discussion about what they’ve been reading about and chat to one another…” – IP2

## Discussion

The experiences of students and educators that participated in the first iteration of an introductory AI module for radiographers (Table 1) were explored.

Overall, participants found the module useful for their learning and practice, but also highlighted points for further improvement. Their experiences were influenced by their role (academic, clinical practitioner or researcher), radiographic modality, level of prior studies, country of residence and practice, local guidance and regulation for the application of AI. Based on their experiences, participants outlined the characteristics of an ideal introductory AI module for radiographers. The following topics (highlighted as subheadings below) need to be considered when designing AI courses for medical imaging professionals, including radiographers.

### Contextual nature of learning experiences

Car et al. [50] found that learners’ social background, needs, knowledge and skills related to education programmes can affect their experiences and attitudes they hold. Our study indicates a similar finding insofar that the professional and educational backgrounds of the participants and their views of what learning and teaching in an introductory module entails were a significant contributor to their experiences of the module evaluated in our study.

### Content-specific recommendations and interprofessional nature

The need to focus on the fundamentals of AI, its clinical applications, data privacy and ethical considerations, as well as algorithmic validity and explainability were deemed important for clinical practice [14, 19, 24, 34]. While participants acknowledged that many of these areas were already taught in the module of this study, they felt more teaching and discussion on the topics of AI evaluation, validation and regulation would be useful. Students also supported the need to empower medical imaging professionals to critique AI-related proposals and applications, as part of the learning, while acknowledging the strong evidence-based approach of the module This is vital since more than half of the respondents in a recent UK study indicated that radiographers lack adequate knowledge about AI and its practical applications [24].

Due to the nature of AI and its implications for medical imaging and radiation sciences professions, interprofessional educational approaches are recommended by participants to foster teamwork and preparation for the workflow changes that will be brought about by AI [51]. Participants highlighted that due to rapid advancement of AI, there is a need to introduce AI at undergraduate level so that students are adequately prepared for the work environment [52, 53].

### Flexibility, blended delivery and adaptability as a requirement for success

This module incorporated both synchronous tutorials and asynchronous discussion boards and time set aside for self-study on the virtual learning management system, where students had another opportunity to engage with their peers and module lead. The participants found the flexibility and synchronous-asynchronous blend of delivery of the module beneficial as it allowed them to participate without major interference with their work schedules. The way the content and module were organised, presented and assessed were all factors that positively contributed to the student participants’ learning, inclusive of the guidance and support offered by the faculty members and module lead. These views expressed by the participants are documented as critical factors for student success in online learning, which increases motivation to learn and engage with the content and learning activities [50, 54–56]. The positive experiences may also be ascribed to the evidence-based approach used to develop and present this module based on previous studies and recommendations published [18–20, 31–34]. Inevitably, with a culturally, educationally and professionally diverse group of students, it may have been hard to meet everyone’s preferences, but modularity and flexibility moving forward would be a strengthening factor of this and any other similar course.

### The implications of the coronavirus pandemic for clinical education delivery

This was the first occurrence of the module, coinciding with the coronavirus pandemic associated restrictions, requiring online only delivery. Student participants problematised learning and teaching in the online space. This sentiment is echoed in the existing body of evidence [54, 55] and it has been a prominent point of discussion about online teaching challenges in forming strong bonds, while trying to adhere to coronavirus pandemic related restrictions [57, 58]. Isolation due to online learning was perceived as a hindrance to student participants’ learning and this is a known factor of attrition, indicated in the literature. Thus, it was deemed important to incorporate activities for a stronger sense of belonging and community [58]. A voluntary student-led synchronous videoconference discussion was suggested as a way to overcome isolation and to extend and reinforce social learning if online delivery is used. Social presence and communities of practice are important to encourage active learning and enhance student success in online programmes [58]. Participants, as clinical practitioners on the frontline, were also, at the time of delivery of this course, overwhelmed with clinical and academic work, so balancing their learning while addressing some overburdening workloads might have impacted their learning experiences and preferences [59].

### Cost, recruitment and sustainability

The cost and limited information provided in the module advertising were highlighted as potential challenges for future recruitment; students felt more could be done to exemplify the module’s unique selling points and extent and depth of learning, to ensure adequate recruitment and future sustainability. The general lack of funding for postgraduate studies is a major challenge for recruitment, in general [60]. This is a frequent challenge experienced by programme directors and should be foregrounded during the planning phases of similar modules or courses. This will assist in managing expectations during the recruitment and marketing phases [54, 60, 61]. With different regulatory and professional bodies advocating for the need of more training on AI as part of the core competencies for radiographers [62] and with more government support to subsidise these AI courses for workforce development and training on new technologies, the number of AI courses in the coming years should increase exponentially.

### Feedback integration in future occurrences

For this occurrence of the module, online learning was a necessity due to the COVID-19 pandemic; subsequent occurrences have been delivered on-campus, in-person, as per original planning, but ideas for online or blended delivery to reach a wider audience should be explored. Many of the above recommendations are now already integrated into the current programme, including more up-to-date content, to capture the latest developments on AI practice and research. Lastly, there is scope for annual review and evaluation of the module to promote a student-centred approach [50, 54] and to align the curriculum with the latest developments in the field of AI.

## Limitations

The findings of this study are context-dependent since different settings may have different needs and resources available. This work is also time-bound, since the field of AI is fast developing and some findings may not be relevant in years to come. Thus, the findings may not be generalisable, but the recommendations emanating from this work can be used as generic principles to inform AI education provisions for radiographers globally. The first occurrence of the module ran online over 12 weeks, due to the coronavirus pandemic associated restrictions. However, the original instructional design was meant to be on-campus, in-person and delivered over one intense week of teaching. Hence, some of the challenges discussed by this cohort of students might relate to the adjusted, online delivery of the module; these challenges are already addressed in the most recent on-campus occurrences of this AI module, to ensure an optimal learning environment and student learning experience.

## Conclusions and recommendations for future research

AI is an ever-advancing field. Participants identified both enabling and hindering factors to their learning for the module evaluated in our study and they proposed recommendations that should be included for an ideal introductory AI module. This work showed that there are many advantages and disadvantages when using different types of delivery. The module length, cost, and delivery format were highlighted as areas needing improvement to enhance active engagement in the module content and activities, as well as to increase student recruitment and programme sustainability. Additionally, participants highlighted that more content on the appraisal of AI literature and the evaluation, validation and regulation of AI tools should be included in the module. Participants further underscored the need to improve the congruence between the content taught and the assessment requirements, and to allow more flexible learning pathways, so that students can align their learning to their interests. This work thus suggests that a blended learning delivery format, adaptive, customisable content, and contextualised to the intended audience, using an interprofessional approach may be a way forward for similar courses in the future. The findings of our work can assist programme directors in other higher education institutions globally, to develop similar education provisions in AI for radiographers and other medical imaging and radiation sciences professionals. As more programmes will be developed and implemented, their evaluation should be published, so that lessons from different contexts can inform global educational practices and approaches in AI education and continuing professional development.

## Data Availability

Not applicable.

## Abbreviations

AI: Artificial intelligence
COREQ: Consolidated criteria for reporting qualitative research
CPD: Continuing professional development
ECTS: European Credit Transfer and Accumulation System
EMEA: Europe, Middle East and Africa
FGD: Focus group discussion
FGDP: Focus group discussion participant
IP: Student interview participant
IPE: Interprofessional education
PAR: Participatory action research
UK: United Kingdom
